# Development of a senior-specific, citizen-oriented healthcare service system in South Korea based on the Canadian 48/6 model of care

**DOI:** 10.1186/s12877-019-1397-3

**Published:** 2020-01-31

**Authors:** Yoon-Sook Kim, Jongmin Lee, Yeonsil Moon, Hee Joung Kim, Jinyoung Shin, Jae-Min Park, Kyeong Eun Uhm, Kyoung Jin Kim, Jung A. Yoo, Yun Kyoung Oh, Pilsuk Byeon, Kunsei Lee, Seol-Heui Han, Jaekyung Choi

**Affiliations:** 1 Konkuk University Medical Center, 120-1 Neungdong-ro (Hwayang-dong), Gwangjin-gu, Seoul, 05030 South Korea; 2 Yonsei University College of Medicine Gangnam Severance Hospital, 211 Eonju-ro, Gangnam-gu, 06273 South Korea; 3 Konkuk University School of Medicine, 268 Chungwon-daero, Chungju-si, Chungcheongbuk-do 27478 South Korea; 4 Department of Neurology, Konkuk University Medical Center, 120-1 Neungdong-ro (Hwayang-dong), Gwangjin-gu, Seoul, 05030 Korea; 5 Department of Family Medicine, Konkuk University Medical Center, 120-1 Neungdong-ro (Hwayang-dong), Gwangjin-gu, Seoul, 05030 Korea

**Keywords:** Senior, Healthcare service, Korea

## Abstract

**Background:**

In the age of aging, Korea’s current medical delivery system threatens to increase the number of medical and caring refugees. This study attempts to develop an integrated senior citizen-oriented healthcare service system in which daily care, professional care, and rehabilitation are organically organized between medical institutions and local communities, thereby meeting the daily life needs of the elderly and inducing well-being, wellness, and well-dying.

**Methods:**

To develop the integrated healthcare system, data collection and analyses were conducted through a systematic review, literature review, benchmarking, focus group interviews, and expert consultation.

**Results:**

The senior-specific, citizen-oriented healthcare service system developed in this study is designed to screen patients aged 65 or older within 24 h of being admitted, using the Geriatric Screening for Care-10. If there is reason for concern as a result of the screening, further evaluation is performed through assessment. Doctors and nurses create a care plan and a discharge plan based on the results from the screening and assessment. The nurse further uses the screening to monitor the patient’s condition before discharge. Based on the screening results at the time of discharge, a transitional care plan is prepared and provided to elderly patients and/or their families. This process enables a systematic link between medical institutions and community resources, aiming for the continuous management of health issues. It also establishes a multidisciplinary treatment plan that considers patients and their families so that diseases common to the elderly are diagnosed and treated promptly.

**Conclusions:**

The most important issue for the elderly is to be able to live healthily and independently for the rest of their lives through well-being, wellness, and well-dying. The senior-specific, citizen-oriented healthcare service proposed in this study is an integrated medical treatment system for elderly users the implementation of which requires the daily care, professional care, and rehabilitation of elderly members of society to be organically organized according to the role of the patients, their families, and the caregiver.

## Background

Aging is a victory for human development. Life extension is one of the greatest achievements of humankind. As standards in nutrition, hygiene, medicine, healthcare, education, and economic life have improved, the longevity of people has become possible [1]. According to United Nations’ *2015 Revision of the World Population Prospects*, the number of people aged 60 years or older will increase rapidly over the next few decades, and the rate of increase will continue to accelerate. Worldwide, the population of those aged 60 years or older is estimated to increase from 901 million in 2015 to about 1.4 billion in 2030 (a 56% increase) and 2.1 billion in 2050 [1]. In Korea, 39% of the population will be aged 60 years or older by 2050 [1]. The life expectancy of Korean women who will be born in 2030 is estimated to be 90.82 years [2]. A serious consequence of population aging is the increase in occurrence of chronic diseases [3], which already account for 60% of deaths worldwide, as well as half of the deaths due to disability [3]. Chronic diseases are the overwhelming causes of disability and death, regardless of income and age [1, 3, 4].

As the elderly population increases, the primary focus is to reduce the gap between life expectancy and healthy life expectancy, the latter being defined as the average expected years of life in a healthy condition for persons at a given age [5].

Korea has a life expectancy of 82.16 years [6], whereas its healthy life expectancy is 73.2 years [7], indicating that the average unhealthy life period lasts about 10 years. This phenomenon gave rise to the proverb “9,988,234” in Korea, which means that you live to be 99 years old (99) by having a healthy life (88), but fall sick for only 2 or 3 days and then die (234). If people live with a disability for the duration of their extended life expectancy, the demand for medical care will increase.

Therefore, the government of the Republic of Korea has been implementing various policies to maximally promote the health and welfare of the elderly, including: (1) visiting home healthcare services for health risk groups; (2) campaigning for healthy life practices to prevent and control chronic diseases, such as lowering sodium intake; (3) operating “100-year-old health athletic classrooms” in highly accessible spaces, such as senior centers, parks, and school playgrounds, as well as elderly exercise voucher projects; and (4) early dementia screening and treatment management support [8]. However, as elderly people have different health and welfare needs, the proportion of total healthcare insurance expenditures represented by the costs of providing elderly medical care has continued to increase, rising from 32.2% in 2010 to 38.7% in 2016 [9].

Korea’s healthcare delivery system allows patients free choice of hospital due to the absence of a control mechanism. In terms of medical institutions, the system is divided into the first stage (clinic and general hospital) and second stage (tertiary hospital). Patients who can be adequately treated at first-stage medical institutions can freely use second-stage medical resources [10].

Primary healthcare, the backbone of the healthcare delivery system, has shrunk significantly over the last decade; it now constitutes only half of Korea’s entire healthcare coverage. This collapse in the healthcare delivery system has hindered the provision of integrated and cost-effective healthcare services by increasing the burden on the public and undermining relations between doctors and patients. As clinics and tertiary hospitals compete for outpatients, the number of patients in tertiary hospitals is growing so rapidly that it reduces quality time between physician and patient. At the same time, this defect in the healthcare delivery system has led to large-scale patient infections in several large hospitals, including a general hospital [11]. Moreover, Korea’s healthcare delivery system fails to ensure post-acute care facilities to provide community-based or general hospital transition care services.

The National Health Insurance Service has emphasized the need to change the healthcare system into one centered on the community: “If we maintain the same hospital-centered medical system in the age of aging, we will face problems such as medical refugees and caring refugees because the management of medical expenses becomes impossible” [9]. Korean hospitals have been unable to grasp the needs of the elderly when providing disease-centered care; healthcare delivery has operated as a one-way system focused on the provider, rather than on the elderly as the user. However, community-based healthcare systems cannot prevent disease occurrence or progression unless they understand the needs of the elderly.

Recently, the concept of “citizen oriented” has been introduced in Korea as a framework for providing customized services. Studies on citizen-oriented endeavors have been conducted mainly in the areas of information and communication technology [12–14]. In science and technology, “citizen oriented” has been defined as “science and technology to meet the daily life needs of the people and improve the quality of life, such as health, safety, welfare, and improvement of life inconvenience” [15]. In other words, ‘citizen oriented’ refers to services (e.g. medical services, information and communication technology, wearable devices, etc.) that people can use in everyday life without discomfort.

This study defines senior specific, citizen-oriented healthcare as a service system that can achieve well-being, wellness, and well-dying by organizing daily care, professional care, and rehabilitation between medical institutions and local communities, as well as satisfying the daily life needs of the elderly in health, safety, welfare, and living. This means that the elderly can be engaged in well-being, wellness, and well-dying if they are provided with medical services that meet their needs where they are located (e.g. hospital, nursing home, community-dwelling, home care, etc.). In this study, well-being, wellness, and well-dying have been defined as follows. Well-being indicated improving life quality and happiness by maintaining self-care and normal physical functioning for daily needs through life-friendly senior healthcare services. Wellness implied health improvement through a personalized healthcare program for the elderly who are able to function independently in their daily care, professional care, and rehabilitation stages, as well as for the elderly who are partially able to provide their own care and those who need assistance. Well-dying referred to ending life happily and beautifully by minimizing the possibility of accidental death caused by increasing bodily deterioration.

People have various social and medical needs in their everyday life, and fulfilling these needs is the starting point for improving quality of life. Improving the quality of life of the elderly is thought to slow down biological, social, and functional ageing and provide active, healthy, and successful ageing. Additionally, if the healthy life expectancy of the elderly can be increased, thereby reducing the period of disease occurrence, then medical expenses related to increased life expectancy may be reduced. Thus, this study attempts to contribute to gerontology and geriatrics by developing a senior-specific, citizen-oriented healthcare service system based on Canada’s 48/6 Model of Care for Well-being, Wellness, and Well-dying.

## Methods

This study established a senior-friendly hospital (SFH) working group for the development of a senior-specific, citizen-oriented healthcare service system. The SFH working group comprised experts from Konkuk University and Konkuk University Hospital specializing in various fields: clinicians (neurology, rehabilitation medicine, respiratory allergy medicine, psychiatry, head and neck otolaryngology, and family medicine); preventive medicine doctors; nurses from mostly elderly wards, dieticians; pharmacists; and a quality improvement facilitator.

In addition, an external expert group was formed to further review the development of a senior-specific, citizen-oriented healthcare service system, comprising professors of urology and surgery, geriatric specialists, elderly drug experts, health administration practitioners, geriatric nurses, community nurses, and health center directors.

**Fig. 1:**
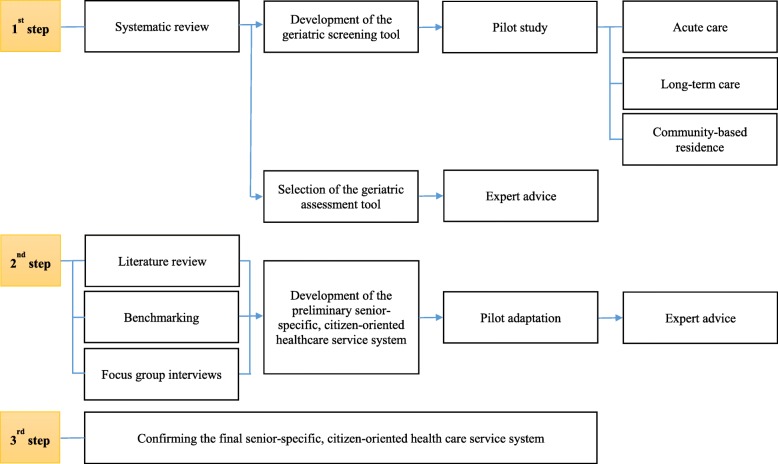
Research process

As shown in Fig. 1, the SFH working group conducted a systematic review and a literature review and benchmarked Canada during the development of a ‘senior-specific, citizen-oriented healthcare service system’. A member of the SFH working group was the moderator for the focus group interviews.

### Step 1: systematic review

To develop the geriatric screening tools and select the geriatric assessment tools, the SFH working group conducted a systematic literature review in accordance with the Preferred Reporting Items for Systematic Reviews and Meta-Analyses (PRISMA) guidelines [16], adopting the following criteria:

- Types of publication: Publications available in English or Korean
- Types of study design: Papers describing original studies, evidence-based guidelines, or systematic reviews
- Types of participants: 65 years or older (with or without cognitive impairment)
- Search strategy: Full-length articles were identified via PubMed, EMbase, Cochrane, KoreaMed, Korean Studies Information Service System, and Korean Medical Database
- Search terms
- Terms covered 10 domains (dementia; depression; delirium; medication [polypharmacy or potentially inappropriate medications or potentially inappropriate prescription]; mobility [mobility or gait or balance]; dysphagia [dysphagia or aspiration]; nutrition [nutrition or malnutrition]; urinary incontinence; fecal incontinence; or pain) and assessment (or scale or measurement or screening or questionnaire or risk factor) and older (or elderly or senior or aged or geriatric) or dementia (or cognitive impairment).

The systematic review proceeded in three steps. First, two reviewers independently reviewed the title, abstract, and original article (excluding duplicates, topics, publication types judged not to fit the inclusion criteria, and publications for which the full-text was unavailable). Then, the articles were reviewed by third-party reviewers to reexamine cases of disagreement between the two reviewers. After settling these disagreements, the final review papers were selected.

An example of a quality assessment for a pain tool development study is presented in Table 1.

**Table 1:** Quality assessment criteria for pain tool

Quality assessment Criteria	Mean score
Explicit theoretical framework	2.7
Statement of aims/objectives in main body of report	2.7
Clear description of research setting	2.7
Evidence of sample size considered in terms of analysis	2.3
Representative sample of target group of a reasonable size	2.1
Description of procedure for data collection	2.7
Rationale for choice of data collection tool(s)	2.7
Detailed recruitment data	2.7
Statistical assessment of reliability and validity of measurement tool(s)	2.6
Fit between stated research question and method of data collection (quantitative only)	2.7
Fit between stated research question and format and content of data collection tool, e.g. interview schedule (quantitative)	2.7
Fit between research question and method of analysis (qualitative)	2.1
Good justification for analytical method selected	2.6
Assessment of reliability of analytical process	2.7
Evidence of user involvement in design (qualitative only)	2.2
Strengths and limitations critically discussed	2.2

Source: Kim YS, Park JM, Moon YS, Han SH: Assessment of pain in the elderly: A literature review. The National medical journal of India 2017, 30(4):203–207

**Fig. 2:**
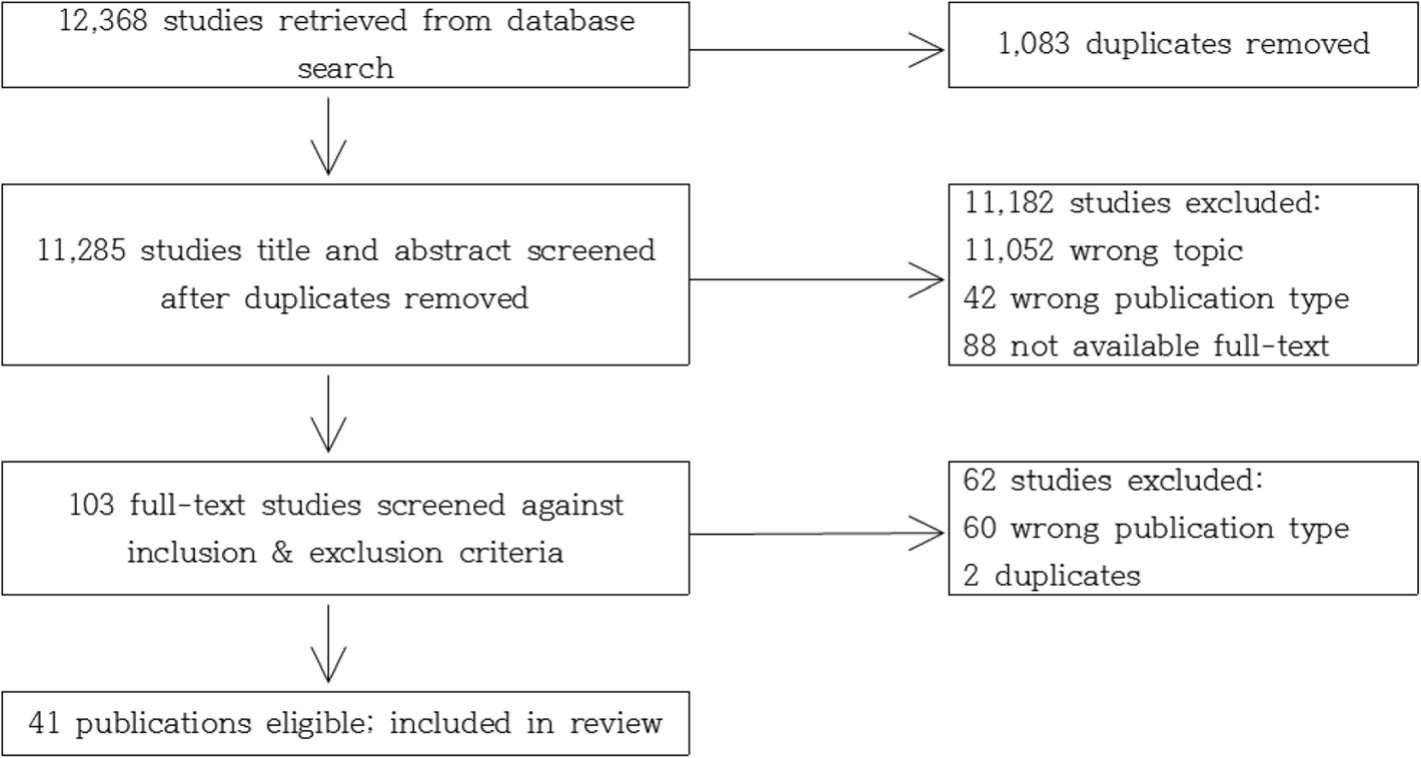
PRISMA flow diagram for the pain tool. Source: Kim YS, Park JM, Moon YS, Han SH: Assessment of pain in the elderly: A literature review. The National Medical Journal of India 2017, 30(4):203–207

The PRISMA flow diagram for the pain tool selection example is shown in Fig. 2.

The other nine domains went through a similar process.

The results of the systematic review are shown in Table 2.

**Table 2:** Results of the systematic review

Domain	Number of initial studies	Number of eligible studies
Cognitive impairment	517	10
Depression	218	57
Delirium	889	32
Polypharmacy	175	7
Functional decline	3746	6
Dysphagia	414	4
Malnutrition	2017	35
Urinary incontinence	2219	140
Fecal incontinence	3302	41
Pain	12,368	41

The aim was to develop a geriatric screening tool that can be used anytime and anywhere to identify possible problems at an early stage. To evaluate the applicability of the tool developed by the SFH working group, a pilot study was performed involving 64 hospitalized patients in acute hospitals, 118 inpatients in long-term care, and 12 elderly people living in the community. Before completing the questionnaire, each individual was given a written explanation of the purpose of the research and they were asked to sign a consent form. Face-to-face interviews were conducted from August 23, 2016, to September 30, 2016, by trained researchers recruited in the study.

The following questions were used in the pilot study:

- Cognitive impairment: declined over the past year?
- Depression: Have you often felt sad or depressed in the last week?
- Delirium: Do you find your current state of consciousness different from usual?
- Medication: Are you currently taking five or more medications? Are you currently attending two or more departments of Medicine (hospitals)? Has your prescription changed within the last month?
- Mobility: Have you had any falls in the last 6 months? Can you move from a bed to a chair/wheelchair? Can you walk to a toilet? Can you climb up stairs?
- Dysphagia: Have you ever (even once) swallowed the wrong way when eating or drinking water in the last 2 weeks?
- Nutrition: Have you lost weight without trying in the last 6 months? If yes, how much?
- Urinary incontinence: Have you experienced accidental urine leakage in the last month?
- Fecal incontinence: Have you experienced accidental bowel leakage in the last month?
- Pain: Have you had pain on more than 1 day in the last 2 weeks?

The pilot study revealed that medical professionals can clearly differentiate between cognitive impairment and delirium, but non-medical professionals, including patients and family members, lack the expertise to identify the differences. The tool for assessing delirium was thus replaced by a Nursing Delirium Screening Scale (Nu-DESC) [17] comprising five items. Nu-DESC is a two-point scale (0–1), with a score range of 0–5; a score of more than 2 points on the Nu-DESC indicated delirium.

The pilot study had the following concerns about medication: Of the total respondents, 45.3% were currently taking five or more medications; 54.7% were currently attending two or more departments of Medicine (hospitals); and 21.9% had experienced a change in their prescription within the last month. Moreover, 70.3% of the elderly respondents answered “yes” to one or more questions, out of the three questions in the medication domain. However, the SFH working group decided to include only polypharmacy in the medication domain of the geriatric screening tool.

Likewise, the tool for screening functional decline was modified for the context of Korean domestic hospitals to refer to the Mobilization for Vulnerable Elders in Ontario (MOVE ON) [18]. On functional mobility, questions concerning experiences of falls were given to all inpatients at the time of admission, which explains its absence from the screening tool.

The dysphagia question was modified, as the original wording “swallow the wrong way” and “once” was found to be too sensitive; the question was modified to “Have you had difficulty in swallowing liquids or foods in the last two weeks?”

We decided to use the Malnutrition Screening Test (MST) [19] at the screening stage to pinpoint the degree of malnutrition. A score of more than 2 points on the MST indicated malnutrition.

The geriatric screening tool derived from the systematic review was named Geriatric Screening for Care-10 (GSC-10) [20]. GSC-10 comprises 10 domains, as shown in Table 3. If a patient had a cognitive impairment and no caregiver, he/she was marked as “not sure.” Rescreening would occur when reliable answers become available.

**Table 3:** The geriatric screening for care-10 (GSC-10)

Domain	Screening question	Answer
Cognitive impairment	Has your relative/friend’s judgment or memory declined over the past year?	□ No □ Yes □ Not sure
Depression	Have you often felt sad or depressed in the last week?	□ No □ Yes □ Not sure
Delirium (Nu-DESC)	Disorientation	□ No □ Yes □ Not sure
	Inappropriate behavior	□ No □ Yes □ Not sure
	Inappropriate communication	□ No □ Yes □ Not sure
	Illusions/Hallucinations	□ No □ Yes □ Not sure
	Psychomotor retardation	□ No □ Yes □ Not sure
Polypharmacy	Are you currently taking five or more medications?	□ No □ Yes □ Not sure
Functional decline	Can you transfer from a bed to a chair/wheelchair?	□ Independent □ Need assistance □ Impossible
	Can you walk to a toilet?	□ Independent □ Need assistance □ Impossible
	Can you climb up stairs?	□ Independent □ Need assistance □ Impossible
Dysphagia	Have you had difficulty in swallowing liquids or foods in the last 2 weeks?	□ No □ Yes □ Not sure
Malnutrition	Have you lost weight without trying in the last 6 months?	□ No □ Unsure
	If yes, how much?	□ Unsure
		□ 1–5 kg
		□ 6–10 kg
		□ 11–15 kg
		□ > 15 kg
	Have you been eating poorly because of a decreased appetite?	□ No □ Yes □ Not sure
Urinary incontinence	Have you experienced accidental urine leakage in the last month?	□ No □ Yes □ Not sure
Fecal incontinence	Have you experienced accidental bowel leakage in the last month?	□ No □ Yes □ Not sure
Pain	Have you had pain on more than 1 day in the last 2 weeks?	□ No □ Yes □ Not sure

*Nu-DESC* Nursing Delirium Screening Scale

The SFH working group selected standardized assessment tools with proven validity. To evaluate dysphagia, the study developed an easy dysphagia symptom questionnaire (EDSQ) that considered the validity of the tool and the situation of medical institutions in Korea. The EDSQ was a 12-item “yes/no” questionnaire on dysphagia symptoms, and the total score was calculated as sum of “yes” responses. Each item was determined by the consensus of three physiatrists after reviewing the dysphagia questionnaires of previous research (Eating Assessment Tool-10 [21], Sydney Swallow Questionnaire [22], swallowing disturbance questionnaire [23], and Kawashima’s dysphagia screening questionnaire [24]). The internal consistency of the EDSQ was assessed by Cronbach’s alpha coefficient, and correlations were analyzed between the EDSQ total score and the scores on other scales, including Modified Water Swallowing Test (MWST), the American Speech-Language-Hearing Association’s National Outcome Measurement System (ASHA NOMS) swallowing scale, and the Videofluoroscopic Dysphagia Scale (VDS). According to Receiver Operating Characteristic (ROC) analysis, the optimal cut-off score was ≥3.5, with a sensitivity of 80.0% and specificity of 67.9%. The diagnostic criteria were ASHA NOMS levels 1 to 3. After consulting with external advisors on the appropriateness of the tool selected by the SFH working group, the study finally selected the following evaluation tools:

- Cognition impairment: Korean-Alzheimer Disease 8 (K-AD8) [25], Mini-Mental State Examination (MMSE) [26], Korean-Montreal Cognitive Assessment (K-MoCA) [27], and Informant Questionnaire on Cognitive Decline in the Elderly (IQCODE) [28].
- Depression: Geriatric Depression Scale-Short Form-Korean (SGDS-K) [29], Korean-Center for Epidemiologic Studies Depression Scale-Revised (K-CESD-R) [30], Patient Health Questionnaire-9 (PHQ-9) [31], and EURO-D scale [32].
- Polypharmacy: Beers Criteria [33], screening tool of older people’s prescriptions (STOPP) criteria, and screening tool to alert to right treatment (START) criteria [34].
- Functional decline: Short Physical Performance Battery (SPPB) [35], Time Up to Go (TUG) Test [36, 37], Performance Oriented Mobility Assessment (POMA) [38], Elderly Mobility Scale (EMS) [39], Berg Balance Scale (BBS) [40, 41], and Hierarchical Assessment of Balance and Mobility (HABAM) [42].
- Dysphagia: EDSQ (draft), Standardized Swallowing Assessment (SSA) [43, 44], water swallow test (WST) [45, 46], and Gugging Swallowing Screen (GUSS) [47].
- Urinary incontinence: International Consultation on Incontinence Questionnaire-Short Form (ICIQ-SF) [48, 49], International Prostate Symptom Score (IPSS) [50, 51], and overactive bladder symptom score (*OABSS*) [52].
- Fecal incontinence: low anterior resection syndrome (LARS) score [53, 54], and Wexner score [55].
- Pain: Numeric Rating Scale (NRS) [56], Visual Analogue Scale (VAS) [56, 57], Faces Pain Scale (FPS) [56, 58], Face-Legs-Activity-Cry-Consolability Scale (FLACC) [59, 60] and Pain Assessment in Advanced Dementia Scale (PAINAD) [61, 62].

The SFH working group provided various evaluation tools to tailor each item for various medical environments in Korea, and made the assessment tool available for selection in individual hospitals.

### Step 2: literature review, benchmarking, and focus group interviews

#### Literature review

The 48/6 Model of Care [63] was reviewed to develop a senior-specific, citizen-oriented healthcare service system for Korea that considers health management and its cost effects with respect to the elderly. The 48/6 Model of Care is an integrated care system that applies a patient-specific care plan within 48 h by screening and assessing six areas of hospitalized elderly patients [63, 64]. It is designed to: 1) identify early the need for treatment in six areas: defecation and urination management, cognitive function, functional mobility, drug management, nutrition and hydration, and pain management; 2) establish individualized treatment programs, including discharge or transition plans; and 3) connect with the community resources needed to improve the quality of life and maintain and improve the functioning of the elderly [63, 64].

#### Benchmarking

The researchers visited the Royal Jubilee Hospital under Island Health in Canada, St. Paul’s Hospital for Providence Health Care, Vancouver General Hospital for Vancouver Coastal Health, and Royal Columbian Hospital for Fraser Health.

#### Focus group interviews (FGIs)

FGI participants included eight elderly inpatients, six family members of patients, and 14 elderly residents living in the community. We included inpatients, caring families, and community-based seniors in the FGIs to develop a tailored, senior-specific, citizen-oriented healthcare service system based on experience in a variety of situations.

The eight elderly inpatients were hospitalised at Konkuk University Medical Centre in an acute phase. They were chosen by head nurses, who explained the purpose and method of the study to them, and they consented to participate.

The six family members of patients comprised family members who were caring for the elderly patients who had been admitted to Konkuk University Medical Centre. The six family members of patients were not the family members of the elderly inpatients who were participating in this study. The family member participants were also selected by the head nurse in the same way as the elderly patient participants had been chosen.

The 14 elderly residents living in the community comprised senior citizens who visit the senior citizen centre daily. They explained the study method and purpose of the director of centre and accepted the study. The head nurse who recruited the study participants was a member of the SFH working group, and the director of the centre was a member of an external expert group.

The FGIs were run by two trained investigators. Each interview session lasted 1 hour. The interview questions were as follows:

- Opening question: For a brief self-introduction, please tell us your name and where you live.
- Introductory question: Tell us how you spend your day (at home/at the hospital/with your sick family member).
- Transition questions: Tell us about your experiences (your visit to the doctor/hospitalization).
- Key questions:
  - ① (When you are sick/when you are with a sick family member) do you feel you need help urgently? If so, please describe what kind of help you need specifically.
  - ② What do you think about (life-friendly) health care services that help you maintain or improve health while causing no discomfort to your life?
  - ③ What do you expect from health care services that help to maintain or improve your health and with no inconvenience to your life?
  - ④ Please describe the roles of hospitals, public health centers, and governments in providing health services that do not inconvenience your life.
- Closing question: Let’s briefly summarize the discussion. Is anything missing? Please tell us if there is something missing or something you would like to add.

In summary, the FGI results showed that:

- The elderly suffered most from depression, pain, side effects of drugs, polypharmacy, functional decline, social isolation and economic problems.
- The families reported that elderly patients who face difficulty with self-care are tired mentally and physically because they are cared for 24 h every day.
- The elderly and their families reported that they needed a system that could immediately provide them with help when they were in emergency situations.
- The state provides considerable support (e.g. medical expenses, financial assistance, etc.) for the elderly aged 65 and over, but the elderly and their families do not believe they are getting any help. The reason for this is that the same services are provided to every elderly person, and the desperate needs of individual elderly patients are not taken into account. Since the elderly face a wide variety of complex problems, they want customised services that account for their personal characteristics.

### Step 3: confirmation through expert consultation

The proposed senior-specific, citizen-oriented healthcare service system, through steps 1 and 2, was confirmed by the SFH working group and external expert committee.

The consultation results of the SFH working group and the external expert committee are as follows.

- A standardised form, such as a transitional care plan, is required to accurately convey information about the health of the elderly during the care transition process.
- In order to prevent the unnecessary waste of resources, a plan must be prepared that can be linked with the elderly services provided by the state.
- It is necessary to suggest management methods for the 10 domains of GSC so that patients and families can engage in self-care, even at home.

## Results

**Fig. 3:**
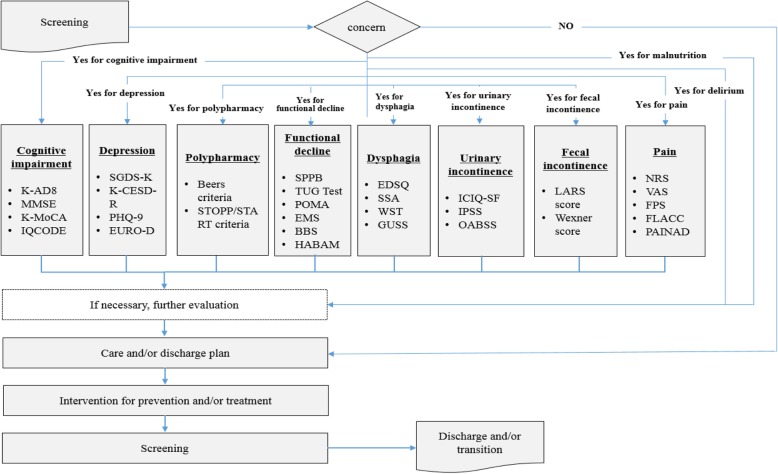
Senior-specific, citizen-oriented healthcare service system. K-AD8, Korean-Alzheimer disease 8; MMSE, Mini-mental state examination; K-MoCA, Korean-Montreal Cognitive Assessment; IQCODE, Informant Questionnaire on Cognitive Decline in the Elderly; SGDS-K, Geriatric Depression Scale-Short Form-Korean; K-CESD-R, Korean-Center for Epidemiologic Studies Depression Scale-Revised; PHQ-9, Patient Health Questionnaire-9; STOPP, screening tool of older people’s prescriptions; START, screening tool to alert to right treatment; SPPB, Short Physical Performance Battery; TUG, Timed Up and Go; POMA, Performance Oriented Mobility Assessment; EMS, Elderly Mobility Scale; BBS, Berg Balance Scale; HABAM, Hierarchical Assessment of Balance and Mobility; EDSQ, Easy dysphagia Symptom questionnaire; SSA, Standardized Swallowing Assessment; WST, water swallow test; GUSS, Gugging Swallowing Screen; ICIQ-SF, International Consultation on Incontinence Questionnaire-Short Form; IPSS, International Prostate Symptom Score; OABSS, overactive bladder symptom score; LARS, low anterior resection syndrome; NRS, Numeric Rating Scale; VAS, Visual Analogue Scale; FPS, Faces Pain scale; FLACC, Face-Legs-Activity-Cry-Consolability Scale; PAINAD, Pain Assessment In Advanced Dementia Scale

Based on Canada’s 48/6 Model of Care, the following senior-specific, citizen-oriented healthcare service system is proposed (Fig. 3).

### Senior-specific, citizen-oriented healthcare service system

#### Purpose of development

The system designed in this study aims to not only identify early the need for treatment in 10 domains relevant to the elderly but also establish an individualized treatment plan that maintains the elderly’s functional status. It also aims to provide necessary resources to the elderly at the time of discharge or transition based on individualized evaluation, to improve the quality of life, health promotion, and safety of the elderly.

#### Development basis

Given the nature of elderly patients, safety-related problems such as delirium, loss of function, falls, and decubitus are twice as high as among young people during hospitalization [65, 66]. In Korea, for hospitalized patients aged 65 years or older, the prevalence of delirium is 5.4 to 19.2% [67], the incidence of adverse drug reactions is 20.2% [68], and the incidence of falls is 42% [69]. In addition, the elderly may experience medical problems during hospitalization owing to changes in function, such as in vision, hearing, cognition, and the musculoskeletal system, caused by aging; further deterioration in underlying diseases or functioning may make it difficult to return to normal daily life [66, 70]. The general pattern of care in Korea is based on patients’ chief complaints. This pattern of treatment is appropriate for young patients who only treat current healthy issues, but not for older people with comorbidity and various potential problems. For the elderly, health problems should be identified at the time of admission, during admission, and at discharge, and they should be provided patient-centered care planning and management. Therefore, it is necessary to change the treatment pattern of the Korean medical institution to ensure that prevention and treatment is provided to the elderly after their overall screening and evaluation.

#### Subjects

A senior-specific, citizen-oriented healthcare service system is applied to hospitalized patients aged more than 65 years, at the stages of discharge and care transition.

#### Screening and evaluation procedures

- Step 1: Hospitalization within 24 h

The nurse will screen elderly patients with the GSC-10 within 24 h of hospitalization. If a concern is identified, the medical staff will evaluate using the tools validated in eight areas (cognition impairment, depression, polypharmacy, functional decline, dysphagia, malnutrition, urinary incontinence, fecal incontinence, and pain). The physician will conduct a further evaluation if necessary, depending on the results of the nurse’s evaluation. If two areas (delirium and malnutrition) are of concern in screening, delirium is further evaluated by the physician as necessary, whereas the malnutrition examination is performed by a nutritionist. The physician will record individualized treatment plans and discharge plans based on the patient screening and evaluation results. Both plans will be recorded even if there is no concern in the patient screening results.

The GSC-10 is intended for all people aged at least 65 years old at the time of admission, and is used to screen for common elderly health problems. The domain of concern in the GSC-10 results is further assessed using a tool for accurate diagnosis.

- Step 2: During the hospital stay

A treatment program is provided according to the patient’s treatment plan. This treatment plan changes as the patient status changes during their hospital stay, and the individualized treatment program is updated accordingly. The patient’s condition is recorded for information sharing across the multiple disciplines providing patient care.

- Step 3: Discharge and/or transition

The SFH working group believed that the elderly should be prepared for discharge from the time of admission to maximize functional mobility even after discharge. After receiving acute care, elderly patients receive ongoing care in sub-acute hospitals, community-based rehabilitation facilities, local nursing facilities, the homes of their family or caregivers, or their own homes. An effective discharge or transition plan is one that maintains the continuity of care by providing information to community-based providers on the resources needed by the patient. It also provides information to caregivers, the elderly, and their families on ongoing care requirements after the patient returns home. To prepare for a successful discharge, the patient and caregiver should accurately understand the patient’s needs. Education and information should be provided to ensure thorough understanding of the necessary medical services available after discharge from hospital.

#### Management

If the GSC-10 identifies a concern at the time of admission, the treatment team, including the patient and caregiver, will address it as outlined in Table 4. In addition, the staff will daily to provide self-care method for the patient and the caregiver. The service plan aims to maintain continuity of care through information sharing between the patient and community resources by establishing patient-specific discharge and/or transition care and action plans.

**Table 4:** Management of concerns

Domain	Principle of management	Management level for provider/patient and/or caregiver
Cognitive impairment	Support optimal cognitive functioning through effective treatment strategies that promote functional independence of all patients, including elderly patients with dementia.	- Early detection and treatment of elderly risk factors (hypertension, diabetes, hyperlipidemia, etc.)
		- Discuss cognitive change in the patient with the multidisciplinary medical team and refer it to the specialist department as appropriate.
		- To address the behavior caused by dementia, first pursue non-medicinal treatment (obesity control, depression management, exercise, and smoking cessation).
Depression	Prevent situations that can cause or exacerbate depression.	- Find medications that aggravate depression early and readjust the medication.
		- Involve the patient, family, and caregiver in a depression care plan, and encourage them to engage in hobbies and exercise with the patient.
Delirium	Prevent situations that can cause or exacerbate delirium.	- Aim for early detection of risk factors in elderly people (dementia, drug changes, hydration, critical illnesses, visual disturbances, environmental changes) to prevent delirium, mediate risk factors, and monitor intervention effects.
		- Educate the patient, family members, and caregiver about delirium, and let them participate in delirium management.
		- Include the management of potential antecedents of delirium (constipation, malnutrition, hydration, urinary catheter, multidrug use, pain, blood sugar elevation) in the multidisciplinary team’s individualized care plan.
Polypharmacy	Reduce the risk of drug interaction. Review medicines to prevent and resolve potential side effects.	- Educate the patient, family members, and caregiver about medicines.
		- Review medicines with pharmacists or physicians, and then develop a sustainable strategy to manage medications the patient is taking.
		- Check drug interactions every time the doctor changes drugs, and ensure there are no drug errors.
		- Re-evaluate and record medicines on a daily basis.
Functional decline	Maintain the patient’s functional athletic ability.	- If it is not medically contraindicated, move as soon as possible after acute illness occurrence.
		- If necessary, provide mobility aids.
		- The use of appropriate shoes is recommended. Consult the patient and caregiver regarding the use of hip protectors.
		- Identify the risk factors for functional mobility loss and personalize multidisciplinary interventions for optimal mobility (e.g., individualized exercise programs).
		- Avoid using restraints. Assess whether the patient’s family can participate in interventions that improve the patient’s ability to move. Include the patient’s family in the planning of the patient’s meals, exercise program, and gait.
		- Provide the patient and their family with information on the risk factors that may limit ability to move.
Dysphagia	Provide safe meals and attitudes to prevent pneumonia caused by dysphagia.	- Monitor malnutrition and airway aspiration due to dysphagia.
		- Identify the current dietary content and provide a diet that can prevent dysphagia.
		- Educate the patient, family members, and caregiver about safe meals and postures.
Malnutrition	Provide proper nutrition. Identify and avoid hospital procedures that cause nutritional imbalance.	- Monitor daily food and water intake, and measure the weight of the elderly.
		- Evaluate the risk factors (dehydration, intravenous fluid, dysphagia, oral illness, delirium) that may affect nutrition and hydration balance.
		- Set goals for food and water intake with elderly patients, and record progress toward achieving goals.
		- Ensure that the patient, family members, and caregiver are part of the nutrition and water management plan and that they can visit to help the patient at meal times.
		- Record the nutrient/ hydration status and plans at transition or discharge.
Urinary incontinence	Maintain urination function through urination management and healthy lifestyle practice. Urinary catheters are used only when medically necessary.	- Ensure the patient is using an appropriate urinary catheter.
		- Consider other methods that can be used instead of urinary catheters (e.g., regular urination training).
		- Evaluate prostate health in male elderly patients.
		- Insert urinary catheters intermittently for urine culture and for management of early urinary obstruction.
		- For patients with symptomatic urinary tract infection, remove urine catheters for longer than 14 days before urine culture. This prevents contamination of the catheter and improves the clinical outcome of antibiotic therapy.
		- Prevent catheter damage and improper removal of the catheter by using a catheter fixation device; this also increases comfort.
		- Indicate symptoms of urinary tract infection to the patient, family, and caregiver.
Fecal incontinence	Maintain normal bowel function through the proper use of regular bowel movements and emollients.	- Check the bowel movement pattern.
		- Perform physical examination and abdominal x-ray.
		- Distinguish temporary incontinence and continuous incontinence.
		- Depending on the degree of constipation, treat with non-medicines and medicines.
		- Provide training to the patient, family members, and caregiver on maintaining the patient’s intestinal health after discharge.
Pain	Assess and manage acute, chronic pain. Identify common causes of acute, chronic pain.	- Implement various non-pharmaceutical approaches as the first intervention for effective pain management.
		- If non-medicinal approaches are inadequate, use appropriate medicines.
		- Drugs should be used at low doses, with increments administered gradually.
		- Start a regularly prescribed dose with a pro re nata (PRN) dose to prepare for sudden pain.
		- When using medicines to manage pain, monitor the side effects of medicines and drug interactions.
		- Monitor and evaluate all pain management interventions.
		- Allow the patient to manage the pain by him/herself.
		- Provide the patient, family members, and caregiver with education on pain.

## Discussion

According to the Korean Medical Law, primary medical institutions, such as medical clinics, serve as gatekeepers for healthcare systems, responsible for the prevention of disease, continuous healthcare, and strengthening of community health. The hospitals are equipped with a medical delivery system for intensive treatment of severe and emergency patients [71, 72]. However, in reality, the medical delivery system is not completely settled, and owing to the competition between general hospitals and clinics over outpatients, patients do not have adequate access to subacute hospital care, primary care, home care, and long-term care after acute care.

Moreover, when patients and/or caregivers require care transition (subacute hospital care, primary care, home care, and long-term care), their current hospital may provide limited information. Consequently, the continuity of medical treatment is disrupted because the care transition plan is not established and evaluated.

This study developed a senior-specific, citizen-oriented healthcare service system, based on the need to establish and manage treatment plans for patients using standardized screening tools. These tools can be used to check for concerns after discharge, and whether the patient chooses to receive ongoing care in a sub-acute hospital, community-based rehabilitation facility, local nursing facility, their family or caregiver’s home, or their own home.

In contrast to the Canadian 48/6 Model of Care [63, 64], the proposed senior-specific, citizen-oriented healthcare service system can provide screening within 24 h to establish a treatment plan. The system applies the Korea Institute of Healthcare Accreditation’s standards for assessing a patient’s condition within 24 h and establishing a treatment and discharge plan [73]. In addition, while the Canadian 48/6 Model of Care [63, 64] comprises six domains (bowel and bladder management, cognitive functioning, functional mobility, medication management, nutrition and hydration, and pain management), the proposed system for Korea comprises 10 domains (cognitive impairment, depression, delirium, polypharmacy, functional decline, dysphagia, malnutrition, urinary incontinence, fecal incontinence, and pain). The SFH working group re-categorized the Canadian domains of bowel and bladder management and cognitive functioning (delirium, depression, dementia), which are regarded as the important physical and mental state changes of the elderly. In the 48/6 Model of Care, dysphagia is included in nutrition and hydration; however, the SFH working group classified dysphagia as an individual domain by regarding it as an important factor in pneumonia and malnutrition. In contrast to the currently used Korean IADL Scale [74] and the Korean Comprehensive Assessment Tools for geriatric ambulatory care [75], the GSC-10 is not limited to any particular assessor (medical team, staff, caregiver, self-report), healthcare setting (acute, long term, community dwelling, or home care), or within-hospital setting (outpatient, inpatient, emergency department).

In Canada, with central government support, each provincial government is responsible for healthcare provision, as well as the operation of its own health insurance plan; all health services are provided to all residents by hospitals, practitioners, dentists, and other medical professionals [76]. In addition, Canada has a cooperative system between community resources for patient transfer or referral, allowing for continuous management after discharge [66]. Korea lacks such a medical delivery system, attributed to reasons such as the shift of patients to large hospitals, excessive input of unnecessarily expensive medical equipment, excessive competition, and polarization of medical institutions. Competition among medical institutions is a costly and wasteful aspect that places importance on reputation, causing qualitative polarization among medical institutions. Consequently, the consumer faces the difficulty of choosing the appropriate medical institution themselves, and ultimately receives segmental, temporary, and fractional services [77]. In 2016, the Ministry of Health and Welfare carried out a pilot project for the transfer or referral of patients between medical institutions to improve the medical delivery system. However, their efforts were limited to improving the delivery system between medical institutions.

When a healthy elderly person is admitted to a medical institution, their psychological and physical condition may not be restored or may even worsen in a system that restricts independent activities [66]. A 2011 report indicated that approximately 30 to 40% of elderly patients lose the ability to perform at least one of their daily activities within 2 days of hospitalization [78]. Meanwhile, about 13% of hospitalized elderly patients die within 1 year of hospital discharge [79]. These facts highlight the importance of not only hospitalization but also continuing care after discharge.

The senior-specific, citizen-oriented healthcare service system was developed to enable continuous management by establishing cooperation between community resources after discharge, as well as during hospitalization. The present healthcare service system applies a medical delivery system to the care transition management between a public health center in the community (subacute hospital care, primary care, home care, and long-term care) and a tertiary hospital to ensure appropriate care transition patient management.

### Strengths and limitations

Senior-specific, citizen-oriented healthcare service systems have been developed through a systematic review, literacy review, benchmarking, FGIs, and advice from various experts. At each stage of the study, we considered which medical and support services are needed for the elderly and their families as well as how to access them in daily life. Thus, the results of this study will be beneficial to stakeholders, including patients and their families, healthcare providers, and government officials. Additionally, this is the first attempt to link medical institutions and community resources in Korea. However, if the proposed system is not implemented by the government, its application would be limited to those hospitals that recognize its merits.

## Conclusions

The most important concern for the elderly is to be able to live healthily and independently for the rest of their lives through well-being, wellness, and well-dying.

The senior-specific, citizen-oriented healthcare service proposed in this study uses an integrated medical treatment system for elderly users. It enables the establishment of patient-specific treatment plans and appropriate intervention through the screening and evaluation of 10 items that organically influence elderly health. The 10 areas are screened not only on admission but also during hospitalization and even after discharge, with an aim of monitoring the health of the elderly patient by observing identified problems even after discharge. In addition, this system enables the prompt diagnosis and treatment of diseases that are common to the elderly through a multidisciplinary treatment plan that can be considered by the patient and their family members. Regarding the issue of the elderly facing heavy social and economic burdens due to aging, one solution is to establish a systematic patient linkage system between medical institutions and community resources.

Since the physician surcharge was abolished in 2018, Korea has seen the proliferation of large hospitals, and its medical delivery system is characterized by clinics and tertiary hospitals competing for outpatients. The government is examining a variety of medical delivery systems, such as a community-based transfer or referral system that can provide appropriate treatments to patients according to the type of medical institution. This study developed the GSC-10, which can be applied in all medical institutions, and an evaluation tool for medical institution-based usage. To facilitate implementation, we also proposed a management method for each item, and presented a model to ensure continuous care for the elderly even after moving to other medical institutions or back into the community.

If the government were to extensively implement the senior-specific, citizen-oriented healthcare service system proposed in this study, the daily care, professional care, and rehabilitation of elderly members of society would be organically organized according to the role of the patient, family members, and the caregiver, ultimately improving well-being, wellness, and well-dying in the elderly.

## Data Availability

The dataset used and analysed during the current study is available from the corresponding author on reasonable request.

## Abbreviations

ASHA NOMS: American Speech-Language-Hearing Association’s National Outcome Measurement System
BBS: Berg Balance Scale
EDSQ: Easy Dysphagia Symptom Questionnaire
EMS: Elderly Mobility Scale
FGIs: Focus group interview
FLACC: Face-Legs-Activity-Cry-Consolability Scale
FPS: Faces Pain scale
GSC-10: Geriatric screening for care-10
GUSS: Gugging Swallowing Screen
HABAM: Hierarchical Assessment of Balance and Mobility
ICIQ-SF: International Consultation on Incontinence Questionnaire-Short Form
IPSS: International Prostate Symptom Score
IQCODE: Informant Questionnaire on Cognitive Decline in the Elderly
K-AD8: Korean-Alzheimer disease 8
K-CESD-R: Korean-Center for Epidemiologic Studies Depression Scale-Revised
K-MoCA: Korean-Montreal Cognitive Assessment
LARS: Low anterior resection syndrome
MMSE: Mini-Mental State Examination
MOVE ON: Mobilization for Vulnerable Elders in Ontario
MST: Malnutrition Screening Test
MWST: Modified Water Swallowing Test
NRS: Numeric Rating Scale
Nu-DESC: Nursing Delirium Screening Scale
OABSS: Overactive bladder symptom score
PAINAD: Pain Assessment in Advanced Dementia Scale
PHQ-9: Patient Health Questionnaire-9
POMA: Performance Oriented Mobility Assessment
PRISMA: Preferred Reporting Items for Systematic Reviews and Meta-Analyses
ROC: Receiver operating characteristic
SFH: Senior-friendly hospital
SGDS-K: Geriatric Depression Scale-Short Form-Korean
SPPB: Short Physical Performance Battery
SSA: Standardized Swallowing Assessment
START: screening tool to alert to right treatment
STOPP: screening tool of older people’s prescriptions
TUG: Timed Up and Go
VAS: Visual Analogue Scale
VDS: Videofluoroscopic Dysphagia Scale
WST: Water swallow test
